# "All Sorts and Conditions" of Women

**Published:** 1889-01-19

**Authors:** 


### The Doctor's Pulpit.

### "ALL SORTS AND CONDITIONS" OF WOMEN.

### The Intellectual Woman.

If an inhabitant of a distant planet should visit this world, and on going up and down in it find out that clever women have still to fight for the liberty of freely using their brains, he would probably not be able to believe the fact. If "custom did not make such dotards of us all," we should be so ashamed of this circumstance in human story, that we should be running into all the railway tunnels and other dark caverns to hide ourselves from our own shadows. In times well within the recollection of middle-aged men, to be called an "intellectual woman" was to be considered an object for commiseration and for some wonder. Not that there have not been such creatures in every age, but they have often played their part in ways so peculiarly feminine, that men have not been aware they were being twisted round the finger and made to dance like puppets by an intellect more subtle and effectual than their own. Nowadays, however, women decline the role of clever, but invisible "wirepullers," and insist that both themselves and their work shall be seen by the audience in exactly the same way as men are seen. The idea that the male human biped should alone, of all God's creatures, be possessed of brains, and the right to use them, is one of those vulgar errors, the survival of which "no fellow can explain." Some people who think themselves mighty clever, not only believe that men, as distinguished from women, possess all the intellect of this world, but all the intellect of all other worlds as well. In the mind of man they will admit there is reason; the vision of before and after; the demand for order and law, purpose, and effectiveness; but outside that brain, though order and law may reign supreme, conscious intelligence there is none. It is wonderful! The self-admiration of man is surely without parallel in any other region of infinite and illuminated space.

Do some men believe in intellectual women? Yea: in intellectual elephants and intellectual octopods too, if they can demonstrate their intelligence convincingly. Intellect is always a "good." If men had a thousand times more than they have they would be all the better. They would be happier too without any doubt. The stupid, stolid, self-satisfied, and brainless world, of all ranks, has always hated and suspected intellect. Not being able to understand, or even to see, what abler men than themselves see, the commonplace multitudes have always treated their intellectual superiors to brickbats and rotten eggs, sometimes of a metaphorical, but not seldom of a material character. Clever women have to suffer in an exactly similar way at the hands of their own sex. It is possible that women of more than average mental power have been worse treated by their fellow-women than men by their fellow-men. Whatever the commonplace and ungenerous woman lacks in brain power she makes up in that most hateful of all qualities, "spitefulness." "Venom" in any form is detested and detestable. The venom of a really spiteful woman's tongue is more poisonous and more vicious than that of the most hideous reptile known. The reptile uses his venom exactly as the lion use his teeth, the bull his horns, or the horse his heels—as a weapon of offence and defence. There is no reason to suppose a snake feels any great concentration of hate when he kills a fat kid, and makes a meal of him. But your really spiteful woman, when once she makes up her mind that you are to be hated, with or without reason, can concoct in her brain, and pour out from her tongue, such a quintessence of viperous poison as might in time turn an archangel into a fiend. Not long ago, at a suburban dinner party, one of the topics of conversation was the recent murder of a wife by her husband. The husband had been a much-enduring man for half a lifetime; the wife had been a conscienceless, spiteful shrew. In a moment of uncontrollable passion the pent-up indignation of years had broken loose, and with one swift but sure blow the long-suffering husband had avenged himself. "She deserved her fate" was the verdict of every woman in the room. Without doubt many clever and noble women have suffered the agonies of martyrdom at the hands of their less intellectual but more spiteful sisters.

What is an "intellectual woman?" A woman of strong sense and right feeling, who exercises her mind freely on all things, great and small. She is not, therefore, a mere scholar. To be able to master languages with some facility, to have a faculty for mathematics or chemistry, implies a certain amount of brain power. But no one will say that every man who knows a little of languages, mathematics, and physical science deserves to be called an "intellectual" man. Similarly a woman must not be considered to have more than ordinary intelligence because instruction and opportunity have given her a smattering of learning. To dub every such woman "intellectual" is to perpetrate a manifest absurdity. Sense, force of character, and right feeling constitute an "able" man. Exactly the same characteristics are the distinguishing marks of the capable woman. It is clear, therefore, that, tried by these definitions, many so-called intellectual woman will be found altogether unworthy of the favourable appellation, whilst many others, whose great and wise energies have been devoted solely to domestic concerns to the almost complete exclusion of academic learning, will take front rank. Can anything be more absurd than to make "able" man or "able" woman identical with "learned" man or "learned" woman? Was the Scottish poet Burns in any sense a learned man? But he was surely an intellectual man. Queen Elizabeth had some of the learning of her times. But was it her learning or her natural strength and capacity of brain which made her the greatest Queen the world then knew? George Eliot was somewhat of a learned woman; but the Brontës were not, nor Mrs. Craik, nor Miss Austen, nor many others whose brains were equal to George Eliot's, though their industry and opportunities were not so exceptional.

Let it not be supposed that there can be any rational objection to learning for women. But mere learning never of itself made either man or woman great. Much sense with little learning will always give a better account of itself than much learning with little sense. By the intellectual woman, we here mean the woman of strong natural brain, of thoroughly honest instincts, and of good heart. Such a woman will always be learned according to her opportunities. If fate has ordained that most of her time shall be spent at the wash-tub, she will wash intellectually and starch rationally, and her work will be stamped with efficiency and success. If she be a teacher of the young she will live leagues away from the house of the pedant, whilst she will not even be on speaking terms with the prig. If novel-writing be her forte and her trade, she will present the reader with men and women whom he can love or hate, fear or despise, imitate or avoid. Puppets on wires and wooden marionettes will not be the work of her hands. Life, reality, truth, and nature will spring from her pen. If she be that noblest, tenderest, and most beautiful of all God's creatures—a wife and a mother, living in and guiding her own home, her soul's simplicity and native strength will find the fittest sphere for growth and development the world can offer. There and nowhere else is the most nobly-intellectual woman to be seen to the greatest advantage. Other women may do well, as well as capacity and opportunity will allow. To what extent they do well, to that extent let them be visited with honours and rewards. But no other woman of equal intellect and character can do so well for herself and the world as the loving and dutiful wife, the tender and guiding mother. Every experienced man will agree with him who said, "Many daughters have done wisely, but she has excelled them all."

*(To be continued.)*

